# The influence of chronic medial epicondylar apophysitis on medial ulnar collateral ligament insufficiency—: a retrospective cohort study

**DOI:** 10.1016/j.jseint.2021.12.014

**Published:** 2022-02-01

**Authors:** Kenichi Otoshi, Shinichi Kikuchi, Kinshi Kato, Yota Kaneko, Ryosuke Mashiko, Ryohei Sato, Takahiro Igari, Takahiro Kaga, Shinichi Konno

**Affiliations:** a Department of Sports Medicine, Fukushima Medical University, Fukushima City, Fukushima, Japan; b Otoshi Orthopaedic Clinic, Oshu City, Iwate, Japan; c Department of Orthopaedic Surgery, Fukushima Medical University School of Medicine, Fukushima City, Fukushima, Japan

**Keywords:** Chronic nonhealed traction apophysitis, Medial epicondyle, High school baseball player, Medial ulnar collateral ligament Insufficiency, Retrospective cohort study

## Abstract

**Background:**

This study aimed to investigate the effect of chronic traction apophysitis of the medial epicondyle (MEC) on medial ulnar collateral ligament (MUCL) insufficiency in high school baseball players.

**Methods:**

In this retrospective cohort study, 3034 of 6069 high school baseball players were enrolled. A self-reported questionnaire was distributed to investigate past history of elbow pain and elbow pain during the previous season. Physical examinations to assess tenderness on the MUCL and the elbow valgus stress test (EVST) were performed. Ultrasonography was performed to determine the presence of morphological abnormalities of the anteroinferior aspect of the MEC (MEC lesions).

**Results:**

Participants with MEC lesions had a significantly higher prevalence of past history of elbow pain, elbow pain during the previous season, MUCL tenderness, and positive EVST than those without MEC lesions (*P* < .05). Multivariate logistic regression analysis revealed that the participants with the fragmented type had the highest risk of past history of elbow pain (odds ratio [OR] = 3.94), elbow pain during the previous season (OR = 2.27), positive EVST (OR = 3.49), and the second highest risk of MUCL tenderness (OR = 2.01) followed by the irregular type (OR = 2.31). Participants with the hypertrophic type had the lowest risk of past history of elbow pain (OR = 2.08), elbow pain during the previous season (OR = 1.42), MUCL tenderness (OR = 1.09), and positive EVST (OR = 1.47).

**Conclusion:**

The presence of chronic non-healed traction apophysitis of the MEC in high school baseball players presented a significantly high risk of elbow pain and MUCL insufficiency.

Traction apophysitis of the medial epicondyle (MEC) is a common elbow injury in adolescent baseball players. The underlying pathology of the lesions associated with apophysitis seems to be due to repetitive traction stress via the medial ulnar collateral ligament (MUCL) to the skeletally immature MEC, as seen in young baseball players.^2^^,^^13^ Various types of morphological changes are observed at the MEC in baseball players, and fragmentation and hypertrophy have been commonly reported.^1^^,^^4^^,^^6^^,^^9^^,^^15^ Adams (1965) reported that 49% of US Little League pitchers had MEC fragmentation,^1^ and Hang et al (2004) reported that the prevalence of MEC hypertrophy and fragmentation was 94% and 19%, respectively.^4^ In a Japanese population, Harada et al (2010) reported that 22% of young baseball players of 9-12 years of age had MEC fragmentation based on ultrasonographic analysis.^6^ Regarding the age-specific prevalence of MEC abnormalities,^15^ the fragmented and irregular types reach a peak at the age of 11-12 years. The occurrence of either type tends to decline with age, whereas the fragmented type remained stagnant at approximately 10% after 14 years of age. On the contrary, the hypertrophic type increases for overlapping reaching more than 50% of all occurrences at 16 years of age. As per this study, there is a high probability that bony avulsion injuries of the MEC might heal, whereas approximately 10% of players have chronic non-healing states at high school age.

It has been reported that fragmented and irregular types could increase the risk of elbow pain and other elbow disorders compared with normal and hypertrophic types in young baseball players.^6^^,^^15^ Furthermore, chronic non-healed traction apophysitis of the MEC, especially the fragmented type, in adult baseball players has been associated with non-responsiveness to conservative treatment, duration of elbow pain, and ulnar neuropathy in skeletally mature players.^3^^,^^19^ In addition, one study revealed that approximately 25% of patients who underwent MUCL reconstruction had MEC fragmentation.^7^ On the other hand, others have demonstrated that the presence of radiographic abnormalities of the MEC in symptomatic players has been variable, and only half of a study population with fragmented-type apophysitis reported a history of soreness.^4^

Because there have been few large epidemiologic studies that investigate the influence of chronic, non-healed traction apophysitis of the MEC on elbow pain induced by MUCL insufficiency, its clinical significance remains unclear, and therapeutic strategy is controversial.^4^^,^^18^ The purpose of this retrospective cohort study was to investigate the influence of residual chronic apophysitis of MEC lesions on elbow symptoms in high school baseball players using data from medical checkups.

## Materials and methods

The present retrospective cohort study involved high school baseball teams from all local communities in Fukushima prefecture in Japan. As per the regulation of Japanese High School Baseball Association, not only official matches, but also practice games are prohibited during the winter period (between December 1 and March 7). Annual medical checkups had been performed immediately after the end of the baseball season. All subjects participated in annual medical checkups conducted during the off-season period between 2014 and 2018.

There were three data collection tools: a self-completed questionnaire, physical examination, and ultrasonographic assessment of the elbow joint. The self-completed questionnaire was predistributed to the subjects and collected on the day of the physical examination. Items on the questionnaire included age, main playing position, total amount of practice per week (days and hours), past history of elbow pain, and the new onset of elbow pain during the previous season. A past history of elbow pain was assessed using the following question: ‘‘Have you ever felt pain or discomfort in your dominant elbow while throwing?”, and the new onset of elbow pain during the previous season was assessed using the following question: ‘‘Did you feel pain or discomfort in your dominant elbow while throwing during the last baseball season?”.

Elbow pain was defined as positive if the player experiences pain during one or more episodes. The physical examination was performed by licensed physical therapists. The presence of MUCL tenderness and elbow pain during the elbow valgus stress test (EVST),^8^^,^^12^ which indicated insufficiency of the MUCL, was assessed on the players’ dominant side. MUCL tenderness was defined as positive if the participants experience pressure pain when the examiner moderately compresses the site between the anteroinferior aspect of the MEC to the ulna sublime tubercle. A positive EVST was defined as the occurrence of pain or discomfort, as perceived by the participants, at their medial elbow during the test.

**Figure 1:**
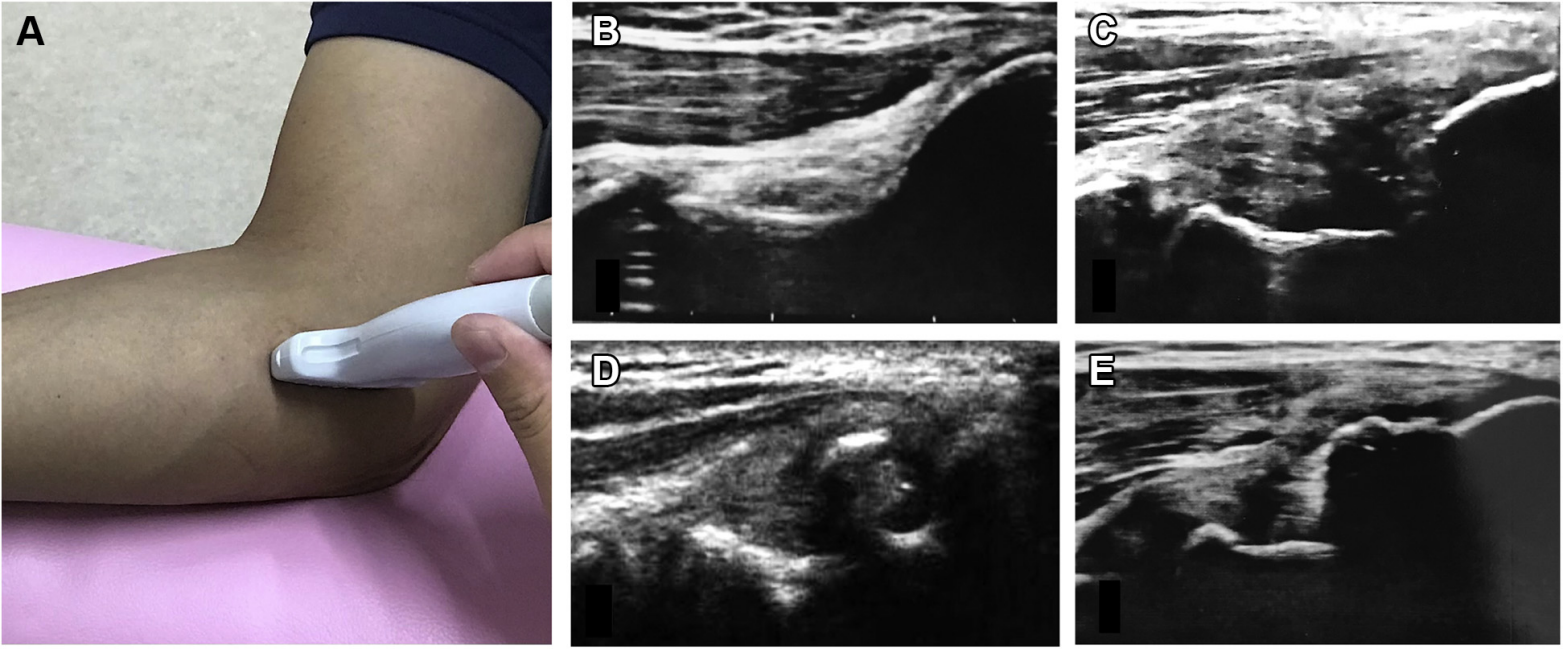
Ultrasonographic examination of the anteroinferior aspect of the medial epicondyle. To assess the medial aspect of the elbow, the elbow is flexed at 90°, and the forearm is placed in the supinated position. A linear transducer is placed on the medial aspect of the elbow to obtain an image that included the *top* of the medial epicondyle, anterior bundle of the medial ulnar collateral ligament, and sublime tubercle (**A**). The morphologies of the medial epicondyle are classified into four types: normal (**B**), irregular type (**C**), fragmented type (**D**), and hypertrophic type (**E**).^20^

**Figure 2:**
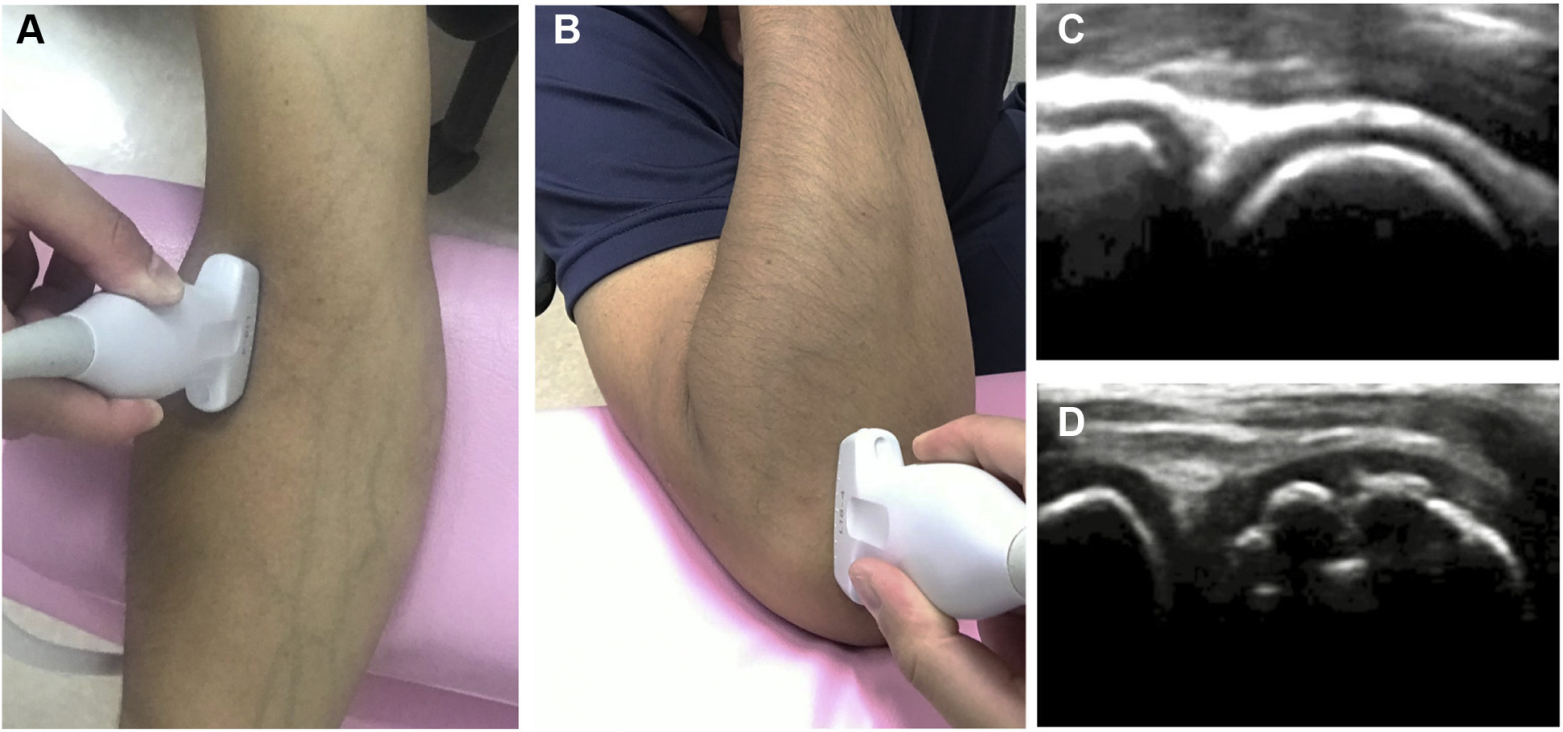
Ultrasonographic examination of the humeral capitellum. To assess the humeral capitellum, the transducer is first placed on the anterolateral aspect of the elbow in the maximally extended position (**A**) and then moved to the posterolateral aspect of the elbow in the maximally flexed position (**B**). The subchondral bone is smooth in the normal capitellum (**C**). Capitellar OCD is diagnosed by irregularity or fragmentation of the subchondral bone of the capitellum (**D**). *OCD*, osteochondritis dissecans.

Ultrasonography was performed on the day of the physical examination to assess any morphological abnormalities of the anteroinferior aspect of the MEC (MEC lesions) and osteochondral lesions of the humeral capitellum (capitellar osteochondritis dissecans [OCD]). Several experienced orthopedic surgeons who received technical training in elbow ultrasonography assessed elbow morphology using the methods described in previous studies.^10^^,^^15^ The morphologies of the MEC were classified into four types: normal (N), irregular (IR), fragmented (FG), and hypertrophic (HT) (Fig. 1).^20^ Capitellar OCD was diagnosed based on the irregularity or fragmentation of the subchondral bone of the capitellum for overlapping (Fig. 2).^10^^,^^15^ We defined the morphology of the MEC and capitellum on the nonthrowing side as normal and compared it with that on the throwing side if determination of the type of morphology was difficult.

The research ethics committee of our institute approved the protocols for this cross-sectional study. All participants provided written informed consent before enrollment.

### Statistical analysis

Participants with complete data were entered into the primary analysis. Continuous data are summarized as mean and standard deviation, whereas dichotomous and categorical data are provided as proportion. The chi-squared test was used to investigate the relationship between MEC lesions with elbow pain, MUCL tenderness, and EVST. Multivariate logistic regression analyses adjusted for age, main playing position, and total amount of practice per week were conducted to investigate the influence of MEC lesions on elbow pain, MUCL tenderness, and EVST. The odds ratios (ORs) and 95% confidence intervals (CIs) were also calculated. Statistical significance was set at *P* < .05.

## Results

**Figure 3:**
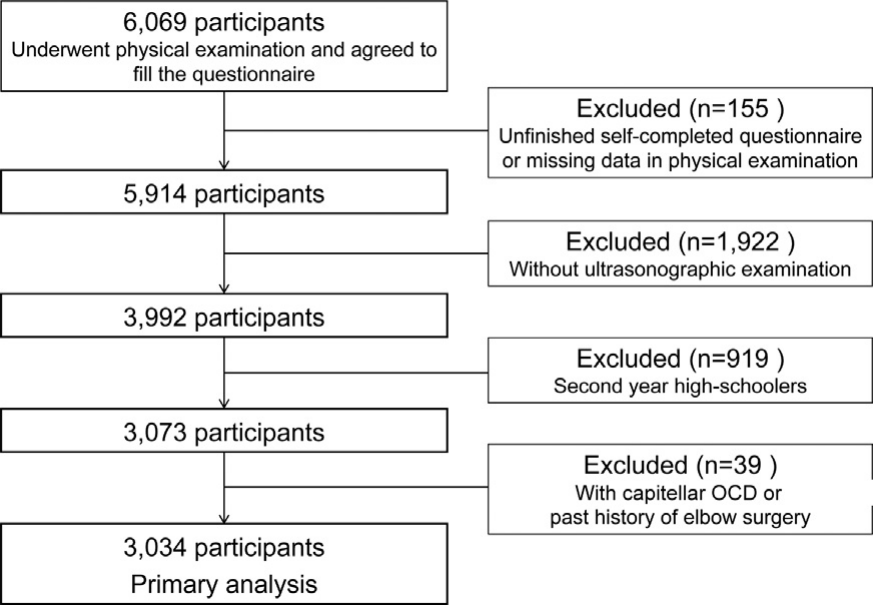
Study flowchart.

A total of 3034 of 6069 high school baseball players were enrolled in this study (Fig. 3). The participants who had not completed the self-completed questionnaire, those with missing data on physical examination (n = 155), and those who had not undergone ultrasonographic examination (n = 1,922) were excluded. To avoid repeated assessments, the participants were limited to first year high schoolers, and over second year high schoolers (n = 919) were excluded. The participants who have experienced elbow surgery on the throwing side and who have capitellar OCD (n = 39) were also excluded.

The mean age of the participants was 15.8 ± 0.4 (range = 15-17) years. Regarding their main playing position, 689 players were pitchers, 294 were catchers, and 2,051 were fielders. During the playing season, 98.9% of the participants played baseball for more than 6 days per week (6 days: 47.3% and 7 days: 51.6%), and the average practice time per week was 26.4 ± 8.3 hours.

### Prevalence of elbow pain, MUCL tenderness, and positive EVST

Of the 3,034 participants, 1,823 (60.1%) had a past history of elbow pain, and 1,195 (39.4%) experienced elbow pain during the previous season. The prevalence of MUCL tenderness and positive EVST was 16.7% (507 participants) and 10.0% (303 participants), respectively.

### Prevalence of MEC lesions

**Figure 4:**
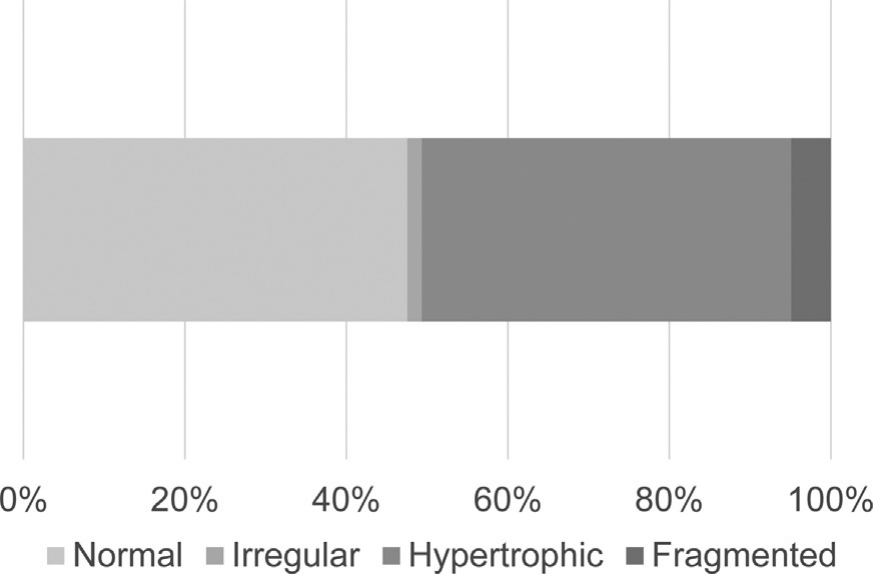
The prevalence of medial epicondyle (MEC) lesions. The prevalence of MEC lesions is 52.6%. Regarding morphology, the hypertrophic (HT) type has the highest prevalence at 45.8%, followed by the fragmented (FG) type at 4.9% and the irregular (IR) type at 1.8%.

Of the 3,034 participants, 1,595 (52.6%) had MEC lesions. The most common morphology was the HT type in 45.8% (1,389 participants), followed by the FG type in 4.9% (150 participants) and the IR type in 1.8% (56 participants) (Fig. 4).

### The relationship between MEC lesions to age, main playing position, and total amount of practice per week (Table I)

As per the univariate analysis, there was a significant association between MEC lesions and the main playing position. Pitchers showed the highest prevalence of MEC lesions (57.8%), followed by catchers (55.1%) and fielders (50.5%). Regarding the type of MEC lesions, the prevalence of IR and FG types was higher in catchers than that in pitchers (IR: 2.4% vs. 1.7% and FG: 7.5% vs. 5.7%, respectively).

**Table I:** The relationship between MEC lesions to age, main playing position, and total amount of practice per week.

	MEC morphology					*P* value
	Normal (n = 1439)	Total MEC lesion (n = 1595)	Each type of MEC lesion			
			Irregular (IR) (n = 56)	Hypertrophy (HT) (n = 1389)	Fragmented (FG) (n = 150)	
Age						
15	365 (50.1%)	364 (49.9%)	16 (2.2%)	310 (42.6%)	37 (5.1%)	
16	1071 (46.5%)	1232 (53.5%)	40 (1.7%)	1078 (46.8%)	113 (4.9%)	.4344
17	3 (75.0%)	1 (25.0%)	0 (0.0%)	1 (25.0%)	0 (0.0%)	
Main playing position						
Pitcher	291 (42.2%)	398 (57.8%)	12 (1.7%)	347 (50.4%)	39 (5.7%)	
Catcher	132 (44.9%)	162 (55.1%)	7 (2.4%)	133 (45.2%)	22 (7.5%)	.0115
Fielder	1016 (49.5%)	1035 (50.5%)	37 (1.8%)	909 (44.3%)	89 (4.3%)	
Total amount of practice per week						
Days	6.5 ± 0.5	6.5 ± 0.6	6.6 ± 0.5	6.5 ± 0.6	6.5 ± 0.5	.2542
Hours	25.7 ± 8.1	27.1 ± 8.5	26.6 ± 8.4	26.9 ± 8.5	28.2 ± 8.4	<.0001

*MEC*, medial epicondyle.

There was no significant association between each MEC lesion and total amount of practice days per week; however, there was a significant association between total MEC lesions and total amount of practice hours per week. Regarding the type of MEC lesions, the FG type was higher than the HT and IR types (28.2 ± 8.4 vs. 26.9 ± 8.5 and 26.6 ± 8.4 hours, respectively).

### Influence of MEC lesions on elbow pain, MUCL tenderness, and positive EVST

Participants with MEC lesions showed a significantly higher prevalence of past history of elbow pain, elbow pain during the previous season, MUCL tenderness, and positive EVST than those without (past history of elbow pain: 69.2 % vs. 50.0%, elbow pain during previous season: 44.3% vs. 34.0%, MUCL tenderness: 18.0% vs. 15.3%, and positive EVST: 12.2% vs. 7.6%, *P* < .05). Participants with the IR and FG type tended to have a higher prevalence of past history of elbow pain, elbow pain during the previous season, MUCL tenderness, and positive EVST than participants with the HT type (*P* < .05) (Table II). To adjust for relevant confounding factors, multivariate logistic regression analyses adjusted for age, main playing position, and total amount of practice per week were performed (Table III). For the MEC lesions, participants with the FG type had the highest risk of past history of elbow pain (OR = 3.94; 95% CI = 2.58-6.23), elbow pain during the previous season (OR = 2.27; 95% CI = 1.59-3.26), and positive EVST (OR = 3.49; 95% CI = 2.19-5.45) and the second highest risk of MUCL tenderness (OR = 2.01; 95% CI = 1.33-3.00) followed by IR type (OR = 2.31; 95% CI = 1.23-4.15). Participants with the HT type had the lowest risk of past history of elbow pain (OR = 2.08; 95% CI = 1.78-2.45), elbow pain during the previous season (OR = 1.42; 95% CI = 1.21-1.66), MUCL tenderness (OR = 1.09; 95% CI = 0.89-1.35), and positive EVST (OR = 1.47; 95% CI = 1.12-1.93).

**Table II:** The relationship between MEC lesions to elbow pain, MUCL tenderness, and positive EVST.

	MEC morphology					*P* value
	Normal (n = 1,439)	Total MEC lesion (n = 1,595)	Each type of MEC lesion			
			Irregular (IR) (n = 56)	Hypertrophy (HT) (n = 1,389)	Fragmented (FG) (n = 150)	
Past history of elbow pain						
Negative	720 (50.0%)	491 (30.8%)	13 (25.0%)	447 (32.7%)	31 (20.7%)	
Positive	719 (50.0%)	1104 (69.2%)	42 (75.0%)	936 (67.3%)	119 (79.3%)	<.0001
Elbow pain during the previous season						
Negative	950 (66.0%)	889 (55.7%)	27 (48.2%)	796 (57.2%)	68 (45.3%)	
Positive	489 (34.0%)	706 (44.3%)	29 (51.8%)	595 (42.8%)	82 (54.7%)	<.0001
MUCL tenderness						
Negative	1219 (84.7%)	1308 (82.0%)	40 (71.4%)	1160 (83.4%)	110 (73.3%)	
Positive	220 (15.3%)	287 (18.0%)	16 (28.6%)	231 (16.6%)	40 (26.7%)	.0009
Elbow valgus stress test						
Negative	1330 (92.4%)	1401 (87.8%)	45 (80.4%)	1242 (89.3%)	116 (77.3%)	
Positive	109 (7.6%)	194 (12.2%)	11 (19.6%)	149 (10.7%)	34 (22.7%)	<.0001

*MEC*, medial epicondyle; *MUCL*, medial ulnar collateral ligament; *EVST*, elbow valgus stress test.

**Table III:** Multivariate analysis for associations between MEC lesions and elbow pain, MUCL tenderness, and positive EVST.

Each type of MEC lesion	Past history of elbow pain		Elbow pain during the previous season		MUCL tenderness		Elbow valgus stress test	
	Odds ratio	95% CI	Odds ratio	95% CI	Odds ratio	95% CI	Odds ratio	95% CI
Normal (N)	1		1		1		1	
Irregular (IR)	3.53∗	1.89-7.10	2.06∗	1.193-.59	2.31∗	1.23-4.15	2.81∗	1.30-5.56
Hypertrophic (HT)	2.08∗	1.78-2.45	1.42∗	1.21-1.66	1.09	0.89-1.35	1.47∗	1.12-1.93
Fragmented (FG)	3.94∗	2.58-6.23	2.27∗	1.59-3.26	2.01∗	1.33-3.00	3.49∗	2.19-5.45

Age, main playing position, and total amount of practice per week were adjusted for logistic regression analysis.

*MEC*, medial epicondyle; *MUCL*, medial ulnar collateral ligament; *EVST*, elbow valgus stress test.

∗ *P* < .05.

## Discussion

This retrospective cohort study aimed to investigate the effect of chronic non-healed traction apophysitis of the MEC on MUCL insufficiency in high school baseball players. Our results indicate that high school baseball players with chronic non-healed traction apophysitis of the MEC are at a significantly higher risk of elbow pain and have positive physical findings indicating MUCL insufficiency such as MUCL tenderness and a positive EVST.

Ultrasonography evaluation of MEC lesions revealed that patients with the IR and FG type had a significantly higher risk of elbow pain and positive physical findings indicating MUCL insufficiency compared with patients with the HT type. Regarding the etiology of each type, it has been reported that the IR type was associated with minimal avulsion injury and the FG type with greater avulsion injury of the MEC. In addition, the HT type was also reported to be indicative of the bony healing of these acute avulsion injuries.^20^ Several studies have demonstrated that bony union was achieved in approximately 90% of the players with fragmented MEC after conservative treatment during their younger age.^5^^,^^11^ However, another study demonstrated that the prevalence of the fragmented MEC had not decreased after 14 years of age and remained stagnant at approximately 10%.^15^ Summarizing these research results, there is a high probability that bony avulsion injuries of the MEC might heal, whereas about 10% of players have chronic non-healing status at their junior high school age, and it would be expected that high school baseball players with the IR and FG types might have chronic non-healed bony avulsion injuries of the MEC that had developed when they were skeletally immature. As our results show that FG and IR types pose a high risk of MUCL insufficiency in high-school baseball players, it is recommended that bony healing of the avulsion injuries should be achieved during their early stages to avoid the aftereffects in adulthood.

Several risk factors for throwing elbow injuries among youth baseball players have been described such as immature skeleton,^5^^,^^11^^,^^14^^,^^16^^,^^17^ and amount of practice has been found to be one of the most important risk factors of elbow disorders. Harada et al (2010) reported that 14 or more hours of training per week and training every day were associated with elbow injuries.^5^ Otoshi et al (2019) also reported that the prevalence of MEC lesions was associated with the total amount of practice per week and duration of the off-season.^14^ As the amount of practice (including the number of throws) and duration of the off-season are among the most important and modifiable risk factors of elbow disorders, controlling the amount of practice and taking an adequate off-season each year might be important for the prevention of traction apophysitis, thereby allowing for bony healing in juvenile baseball players, especially before 14 years of age.

This study has several limitations. The first is the study design. Because this was a retrospective cohort study, the evidence level might not be as high as that of a prospective study. Second, this study only investigated the morphological change of the MEC and did not assess the ultrasonographic abnormality of the MUCL. Furthermore, this study did not confirm other pathogenesis of elbow pain, such as osteoarthritis, ulnar nerve neuropathy, and stress fracture of the olecranon, which may occur as a throwing elbow injury in adults. Therefore, it might be necessary to exclude these coexisting pathologies using imaging studies to certify the involvement of traction apophysitis of the MEC. Third, our study did not evaluate the intraobserver and interobserver reliability. Although we held a pre-examination meeting to confirm the judging criteria and technical pitfall regarding the ultrasonographic assessment to minimize any possible differences among the opinions of the examiners, it is necessary to establish these reliabilities for more precise evaluation.

Finally, our statistical examination might not be sufficient because it did not include all confounding factors associated with elbow pain. Prospective cohort study including relative confounding factors should be conducted to demonstrate the real influence of chronic medial epicondylar apophysitis on MUCL insufficiency.

## Conclusion

Our study revealed that the presence of chronic non-healed traction apophysitis of the MEC in high school baseball players posed a significantly high risk for elbow pain, and positive physical findings indicated MUCL insufficiency. It might be important to reduce the onset of traction apophysitis of the MEC and accelerate bone healing at their youth stage to prevent MUCL insufficiency in their postgrowth.

## Disclaimers

Funding: No funding was disclosed by the authors.

Conflicts of Interest: The authors, their immediate families, and any research foundation with which they are affiliated have not received any financial payments or other benefits from any commercial entity related to the subject of this article.
